# Supplementary material for: Exploiting routinely collected severe case data to monitor and predict influenza outbreaks
Source: BMC Public Health. 2018 Jun 26;18:790. doi: 10.1186/s12889-018-5671-7 (PMC6020250; doi:10.1186/s12889-018-5671-7)
Supplement: Supplementary file 1 — Appendix to the main text, including further explanation of the models and algorithms used, description of the methodological challenges and supplementary results. (PDF 8040 kb) [file 12889_2018_5671_MOESM1_ESM.pdf]

Appendix to the article Exploiting routinely collected severe case data to monitor and predict influenza outbreaks

A. Corbella[†§1], X.-S. Zhang [§2], P. J. Birrell[1], N. Boddington[2], A. M. Presanis[1], R. G. Pebody[2], and D. De Angelis[1,2]

[§]Equal contributors

[1]Medical Research Council, Biostatistics Unit - University of Cambridge, School of Clinical Medicine
[2]Centre for Infectious Disease Surveillance and Control, Public Health England
[†]Author for correspondence: alice.corbella@mrc-bsu.cam.ac.uk

October 17, 2017

Contents

1 Estimation of the Observational model **2**

2 Transmission model **6**
2.1 Definition of the model 6
2.2 Solution in 0 and re-parametrisation 6

3 Challenges of the inference **8**
3.1 The Metropolis Hastings algorithm 8
3.2 Too many parameters: an issue on identifiability 9

4 Supplementary results **11**
4.1 History of our model 11
4.2 Results on the full datasets 12
4.2.1 Results with uniform priors 12

4.2.2 Results with informative priors 17
4.3 Results on the datasets updated every five weeks 21

5 Simulation of a pandemic **22**
5.1 Results on the full datasets 23
5.1.1 Results with uniform priors 23
5.1.2 Results with informative priors 26
5.2 Results on the datasets updated every five weeks 28

Introduction

This document is an appendix to the article *Exploiting routinely collected severe case data to monitor and predict influenza outbreaks*. In Section 1 we report the derivation of the observational model (Equation 5 of the main text). In Section 2 we report the re-parametrization of the transmission model. In Section 3 we report the algorithmic choices and the challenges faced in drawing the posterior distribution of the parameters. In Section 4 we report all the results obtained on the analysis of real data. Lastly, in Section 5 we test our model in a simulated pandemic situation.

1 Estimation of the Observational model

The density function of the time from infection to Intensive Care Unit (ICU) admission plays a crucial role in the computation of the likelihood of the UK Severe Influenza Surveillance System (USISS) data, as can be seen from equation 5 of the main text:

$$\mu_w = \sum_{v=0}^{w} f_{ICU|I}(w-v) \cdot \Delta I_v p_{ICU}.$$

$f_{ICU|I}(w)$ denotes the probability of waiting w weeks between infection and ICU admission. This information, combined with the number of new infections ΔI_v and the probability of ICU admission given contact p_{ICU} allows the computation of the average number of ICU admission at a given week w, denoted by μ_w. The process is represented in figure 1. To obtain an estimate of $f_{ICU|I}(w)$ we considered the incubation time, a random variable denoted by Y, and the time from symptom onset to ICU admission, a random variable denoted by Z.

The random variable Y was estimated by [1] via parametric survival analysis of individual level data, and resulted in the following approximation

$$Y \sim \text{Gamma}(\alpha = 0.678, \beta = 0.417) \tag{1}$$

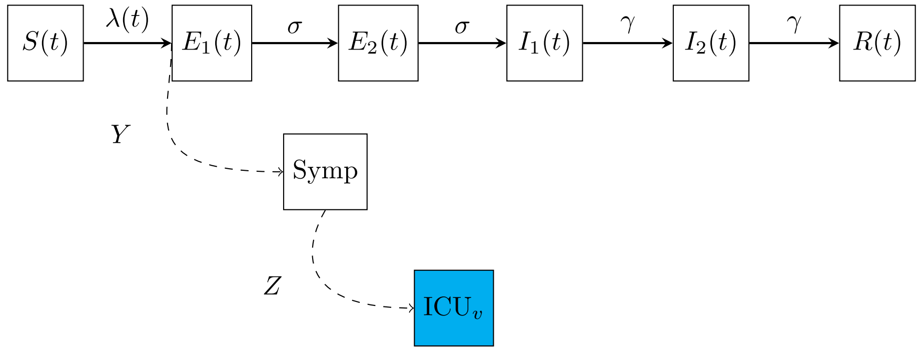

Figure 1: Extended observational model of ICU/HDU admissions. Transmission model is linked to ICU/HDU admissions thought the random variables Y, the incubation time, and Z, the time from symptom to ICU admission.

with $E(Y) = \frac{\alpha}{\beta}, Var(Y) = \frac{\alpha}{\beta^2}$.

We used data form the USISS sentinel scheme to approximate the distribution of Z, the time from symptom onset to ICU admission. The patients admitted to ICU in the sentinel Trusts were asked to report the day of their first symptoms. We fitted several parametric survival curves to the data on 120 patients admitted to ICU during influenza seasons 2011/12 and 2012/13 and selected the model with lowest Akaike Information Criterion (AIC) [2].

The fitted distributions are reported in Figure 2 and the results are reported in Table 1.

	model	par1(SE)	par2(SE)	logL	AIC
1	Exponential	rate = 0.32 (se= 0.028)	/	-280.3059	562.6118
2	Weibull	shape = 0.976 (se= 0.062)	scale = 3.087 (se= 0.293)	-280.2274	564.4549
3	LogNormal	meanlog = 0.607 (se= 0.089)	sdlog = 1.022 (se= 0.063)	-268.2563	540.5126
4	Gompertz	shape = -0.047 (se= 0.023)	rate = 0.377 (se= 0.043)	-277.837	559.6741
5	Gamma	shape = 1.074 (se= 0.118)	rate = 0.344 (se= 0.047)	-280.0966	564.1933

Table 1: Estimates of the parameters and AICs of the parametric models used to fit data on the time from symptom onset to ICU admission.

In conclusion, we approximated Z by

$$Z \sim \text{LogNormal}(\mu_{\log} = 0.607, \sigma_{\log} = 1.022). \tag{2}$$

The density functions of the two random variables X and Y are reported in figure 3.

To compute $f_{ICU|I}(w)$ for week $w = 0, 1, 2, \ldots$ we firstly determined the cumulative distribution of the random variable T, the time in days from infection to ICU admission

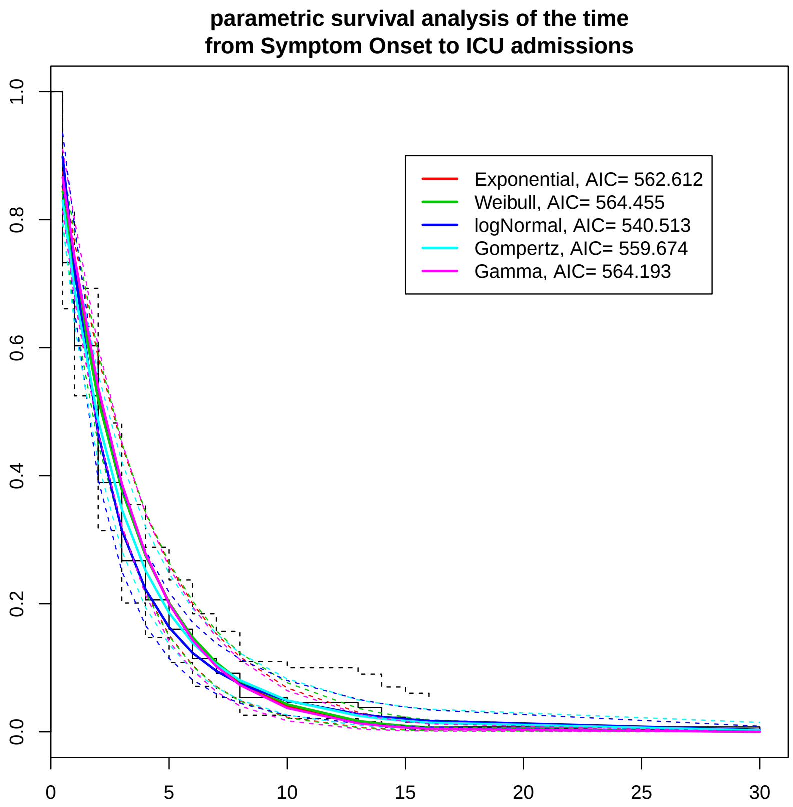

Figure 2: Fitted parametric distributions to the time form symptom onset to ICU admission. Dotted lines represent 95% Confidence Intervals

as the convolution of Y and Z. This is reported in Equation 3. Given a random variable X we use common notation and we denote its cumulative distribution function by $F_X(x) = \mathrm{P}(X \leq x)$ and its probability density function by $f_X(x) = \mathrm{P}(X = x)$.

$$F_T(t) = P(T \leq t) = \int_{y=0}^{t} f_Y(y) \cdot F_z(t-y)dy \tag{3}$$

By numerical integration we compute $F_T(t)$ for $t = 7, 14, 21, \ldots$ so that the probability of w week elapsing from infection to ICU admission can be simply calculated using equation 4 and its values for small number of weeks are reported in Table 2.

$$f_{ICU|I}(w) = F_T(w \cdot 7) - F_T((w-1) \cdot 7) \tag{4}$$

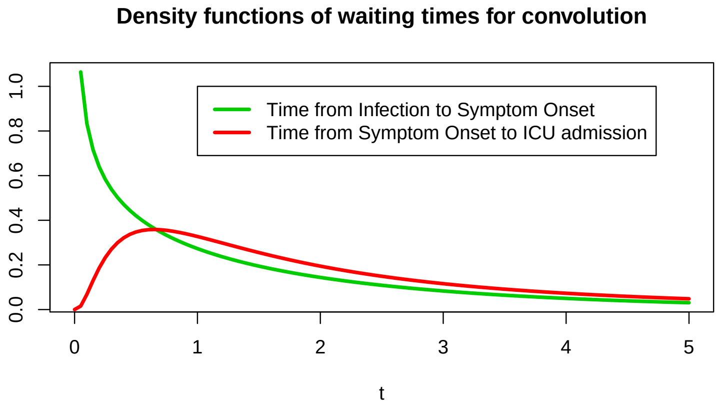

Figure 3: Estimated density function for the incubation time Y and the time from symptoms to ICU admission Z.

week	$f_{ICU\|I}(w)$
1	0.81128
2	0.15180
3	0.02546
4	0.00670
5	0.00242
6	0.00106
7	0.00052
8	0.00028
9	0.00016
...	...

Table 2: Estimated probability function of the convolution over weeks of the incubation time Y and the time from symptom onset to ICU admission Z.

2 Transmission model

2.1 Definition of the model

We used a deterministic epidemic model in continuous time, defined by the following system of differential equations.

$$\begin{aligned}
\frac{dS}{dt} &= -\lambda(t) \cdot S \\
\frac{dE_1}{dt} &= \lambda(t) \cdot S - \sigma \cdot E_1 \\
\frac{dE_2}{dt} &= \sigma \cdot E_1 - \sigma \cdot E_2 \\
\frac{dI_1}{dt} &= \sigma \cdot E_2 - \lambda \cdot I_1 \\
\frac{dI_2}{dt} &= \lambda \cdot I_1 - \lambda \cdot I_2 \\
\frac{dR}{dt} &= \lambda \cdot I_2
\end{aligned} \tag{5}$$

During the epidemic, the infection rate $\lambda(t)$ is determined by the transmission rate, denoted by $\beta^*(t)$, and the proportion of infectious people in the whole population according to the law of mass action [3] (Equation 1 of the main text).

Where $\beta^*(t)$ is equal to $0 \leq \beta\kappa \leq 2 \cdot \beta$ during school closures and to β otherwise.

We can derive the basic reproduction number R_0, i.e. the average number of successful transmissions for a typical infectious person in a fully susceptible population.

$$R_0(t) = \beta^*(t) d_I \tag{6}$$

If we assume that only a proportion π is susceptible at $t = 0$ (e.g. because of vaccination or acquired immunity), we can derive the effective reproduction number R_n, i.e. the average number of successful transmissions per infectious person in a partially susceptible population.

$$R_n(t) = R_0(t)\pi \tag{7}$$

All the parameters that depend on $\beta^*(t)$ inherit its piecewise constant shape. The transition rates, together with assumptions on the state of the compartments at $t = 0$ $(S(0), E_1(0), \ldots R(0))$, completely determines the evolution of the population through system 5.

2.2 Solution in 0 and re-parametrisation

The epidemic model is set to start at $t = 0$. If $t = 0$ is set (on the calendar time) to be close enough to the real emergence of the epidemic in the population, then we can set as an initial condition that the compartment size E_1, E_2, I_1 and I_2 are growing exponentially with rate ψ. Note that we are not making an assumption on the dynamics of the system over the epidemic, instead, we are adopting a re-parametrization in order

to reduce the number of parameters to set or to estimate. In fact, beside the transition parameters (β, κ, σ, γ), also the initial state of the system ($S(0)$, $E_1(0)$, $E_2(0)$, $I_1(0)$, $I_2(0)$, $R(0)$) is to be found or set. This paragraph explain the re-parametrization that allows us to reduce the dimension of the parameter space.

When the epidemic is at its start ($t = 0$), we set the compartment size E_1, E_2, I_1 and I_2 to follow an Exponential Growth Rate (EGR) dynamic ([3]) as shown in Equation 8.

$$\begin{aligned}\frac{dE_1}{dt} &= \psi \cdot E_1 \\ \frac{dE_2}{dt} &= \psi \cdot E_2 \\ \frac{dI_1}{dt} &= \psi \cdot I_1 \\ \frac{dI_2}{dt} &= \psi \cdot I_2\end{aligned} \tag{8}$$

We can equate each line of System 8 with the respective equations of System 5:

$$\begin{aligned}\lambda(0) \cdot S(0) - \sigma \cdot E_1(0) &= \psi \cdot E_1(0) \\ \sigma \cdot E_1(0) - \sigma \cdot E_2(0) &= \psi \cdot E_2(0) \\ \sigma \cdot E_2(0) - \lambda \cdot I_1(0) &= \psi \cdot I_1(0) \\ \lambda \cdot I_1(0) - \lambda \cdot I_2(0) &= \psi \cdot I_2(0)\end{aligned} \tag{9}$$

To solve this system we introduce the quantity I_0^{tot} defined in the main text as the total number of infectious individuals at $t = 0$ (i.e. $I_0^{\text{tot}} = I_1(0) + I_2(0)$). The solution of Equation 9 is reported in Equation 10, where the first and last line are derived by assumption of closed population and definition of initial immunity $(1-\pi)$, respectively.

$$\begin{aligned}S(0) &= N - I_1(0) - I_2(0) - E_1(0) - E_2(0) - R(0) \\ I_1(0) &= I_0^{\text{tot}} \frac{1}{1 + \frac{\gamma}{\gamma+\psi}} \\ I_2(0) &= I_0^{\text{tot}} - I_1(0) \\ E_2(0) &= I_1(0) \left(\frac{\gamma + \psi}{\sigma} \right) \\ E_1(0) &= E_2(0) \left(\frac{\sigma + \psi}{\sigma} \right) \\ R(0) &= (1 - \pi)N\end{aligned} \tag{10}$$

This result was firstly derived in [4] and further used in [5] to model the spread of influenza in UK. For this reason it seems a sensible re-parametrisation for the data we are analysing to reduce the number of parameters of our system.

[4] proved that, under this parametrisation, the basic reproduction number R_0 can be expressed as a function of the rate ψ, of the average infectious period d_I, and of

the average incubation period d_L as in Equation 11.

$$R_0 = \psi \cdot d_I \cdot \left\{ \frac{\left(\frac{\psi d_L}{2} + 1\right)^2}{1 - \left(\frac{\psi d_I}{2} + 1\right)^{-2}} \right\}. \quad (11)$$

We can further re-parametrize by defining a new parameter $\lambda_0 = \lambda(0)$, the hazard of infection at the beginning of the epidemic:

$$\lambda_0 = \beta^*(0) \cdot I_0^{\text{tot}}. \quad (12)$$

Rewriting 12 as a function of λ_0 and I_0^{tot} and combining this with Equation 6 we can write I_0^{tot} as a function of the other parameters.

$$I_0^{\text{tot}} = \frac{d_I \lambda(0) N}{R_0}. \quad (13)$$

In conclusion, the transmission model is defined by the parameter vector $\mathbf{\Theta} = (\lambda_0, \psi, \pi, \sigma, \gamma, \kappa)$ and using Equations 6 to 13 we can derive the initial states, the transmission rate $\beta^*(t)$ and the basic and effective reproduction number R_0 and R_n.

3 Challenges of the inference

3.1 The Metropolis Hastings algorithm

We formulated a Metropolis-Hastings (MH) algorithm [6] to sample from the posterior distribution of $\mathbf{\Theta} = (\pi, \lambda_0, \psi, \eta, p_{ICU}, \kappa)$. Values were proposed by sampling from a truncated log-normal random walk[1]. The elements of $\mathbf{\Theta}$ were very correlated (see results on identifiability in Section 3.2): this was diagnosed while running the MH algorithm on each element of the vector $\mathbf{\Theta}$, conditional on the others. $\mathbf{\Sigma}$, the variance covariance matrix of the parameter vector, was estimated by $\widehat{\mathbf{\Sigma}}$, the observed variance covariance matrix of the sampled values of $\mathbf{\Theta}$.

This matrix is used to formulate a blocked MH algorithm that allows the simultaneous sample and update of all the elements of the vector together [7]. In the blocked MH algorithm, a single sample is drawn from multivariate log-normal distribution for all the elements of $\mathbf{\Theta}$:

$$\mathbf{\Theta}^* \sim LN(\mathbf{\Theta}, \widehat{\mathbf{\Sigma}}\nu) \quad (14)$$

where $\widehat{\mathbf{\Sigma}}$ is the variance-covariance matrix of the samples obtained from the conditional version of the algorithm (i.e. when each element of the vector $\mathbf{\Theta}$ is sampled conditional on the other elements).
The proposed $\mathbf{\Theta}^*$ is accepted with probability ρ, a function of the prior $(\pi(\mathbf{\Theta}))$, the

[1]A log-normal random walk is a discrete time continuous space stochastic process formed of conditional samples from log-normal distributions. In our case, since the space of the parameters is bounded, the random walk has to be constraint into these bound, and this is obtained by truncating the log-normal random variables we sample from.

likelihood ($p(\mathbf{y}|\mathbf{\Theta})$) and the transition probabilities ($q(\mathbf{\Theta}^*|\mathbf{\Theta})$, $q(\mathbf{\Theta}|\mathbf{\Theta}^*)$, i.e. the density function of the proposal value) as formulated in Equation 15.

$$\rho = \frac{\pi(\mathbf{\Theta}^*) \cdot p(\mathbf{y}|\mathbf{\Theta}^*) \cdot q(\mathbf{\Theta}|\mathbf{\Theta}^*)}{\pi(\mathbf{\Theta}) \cdot p(\mathbf{y}|\mathbf{\Theta}) \cdot q(\mathbf{\Theta}^*|\mathbf{\Theta})} \tag{15}$$

Finally, ν denotes the tuning parameter to allow good mixing of the sampled chain [7].

The algorithm used is available at the web address `http://www.mrc-bsu.cam.ac.uk/software/miscellaneous-software/`.

3.2 Too many parameters: an issue on identifiability

As introduced above and in the main text, the distribution of the parameters π, ψ and p_{ICU}, conditional on the full data, are highly correlated. When we further explored the causes of this by investigating the likelihood, we detected regions (i.e. combination of the parameters) where the log-likelihood was equally maxima (these correspond to the darkest regions of Figure 4). Locally flat likelihoods, such as those obtained in our study, proved to be a cause of lack of identifiability [8], this means that only some combinations/functions of these parameters can be estimated.

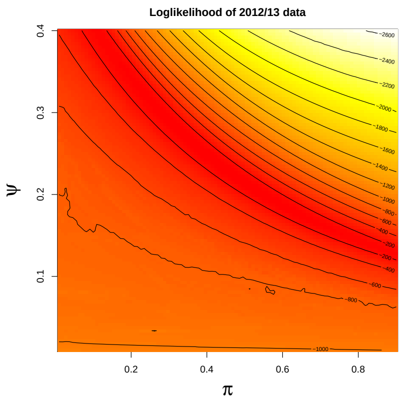

Figure 4: Contour plot of the log likelihood of the full dataset from season 2012/2013 as a function of the parameters ψ and π. All the other parameters were set to the median values reported in Table 3.

Parameters are even more affected by this problem when fewer data are available (i.e. at the beginning of an epidemic). This can be visualized in Figure 5: here we plot the two-dimension posterior samples from the parameters as the dataset is updated. The lighter points in yellow represent the samples of the parameters when data up to week 3 are available, and, increasingly, the more information is acquired the darker color denotes the posterior sample. Other couples of parameters (e.g. p_{ICU} and κ) were initially very correlated but, as data arrive, they acquire their own distribution which is not determined by the other one any more.

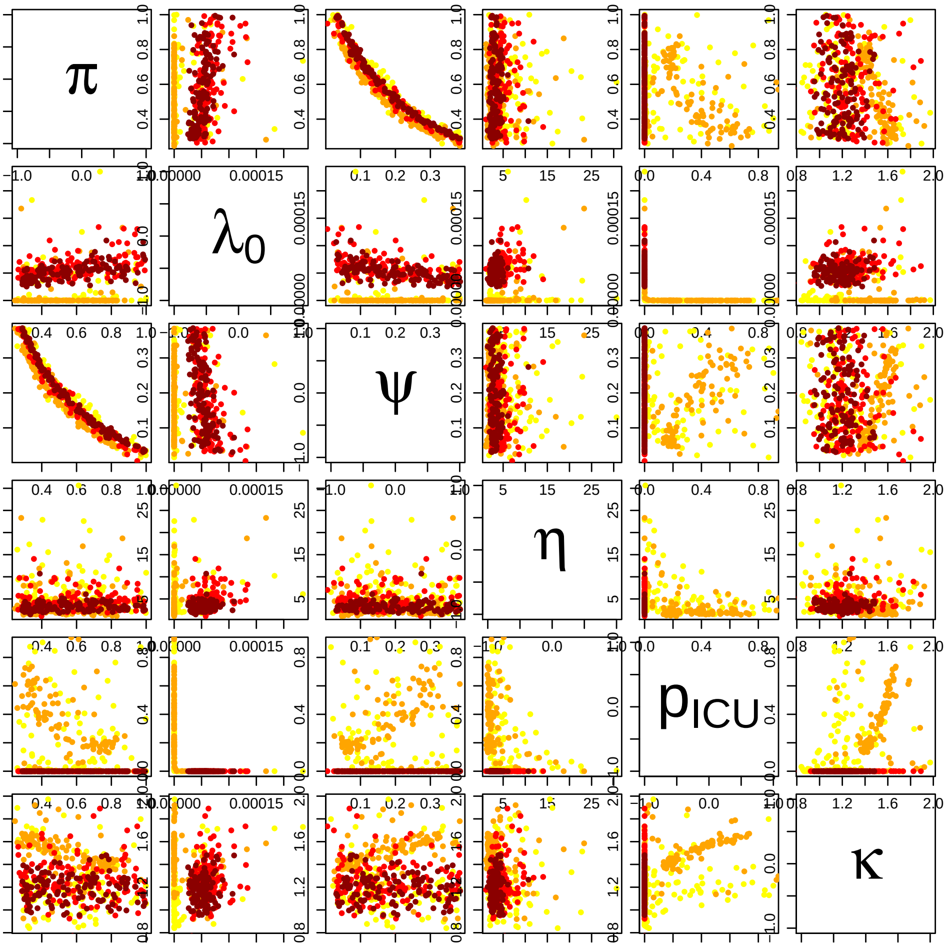

Figure 5: Bivariate scatter plot of a sub-sample of the posterior drawn of all the parameters as the data available progress. Darker points are obtained using more data.

In Bayesian reasoning, the other method to improve the posterior distribution is to inject information via the definition of less-variable prior distributions. The same scatter plots as before, within this informative scenario are reported in figure 6, where the constraints imposed by the data (i.e. the high correlation) are combined with independent informative priors on the parameters π and p_{ICU}.

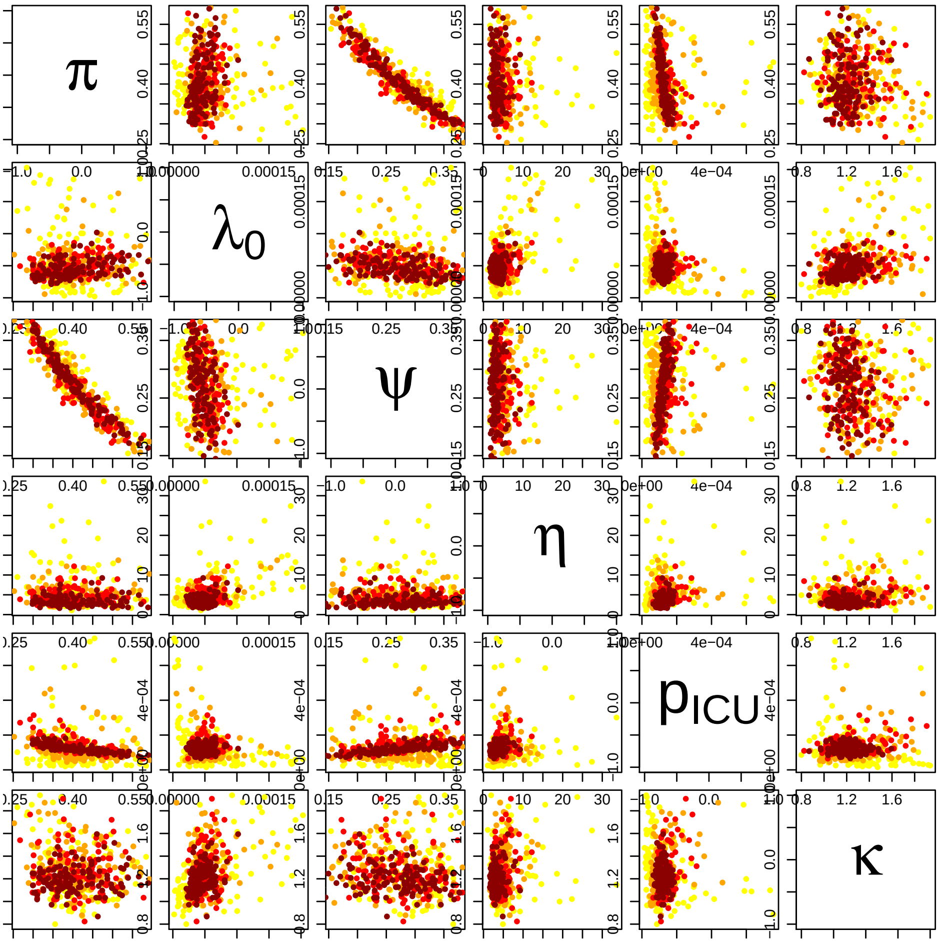

Figure 6: Bivariate scatter plot of a sub-sample of the posterior drawn of all the parameters as the data available progress. Darker points are obtained using more data.

4 Supplementary results

4.1 History of our model

The results reported in the main text and in this section were obtained using the model explained in the main text and in section 3 of this document. However several models were tested before selecting the optimal model.

Specifically, we started from a discrete-time model evolving on quarters of days, but then we preferred a continuous-time model to eliminate one level of temporal approximation and to reduce computational time.

Moreover we initially assumed a transmission rate constantly proportional to the number of infectious:

$$\lambda(t) = \beta I(t) \tag{16}$$

and we decided to allow for the scaling factor κ after having noted a the poor fit of our model to the double peaked data of season 2014/15.

Lastly we attempted several re-parametrisation to address the identifiability issue explained in Subsection 3.2. Despite other parametrizations of the model were competitive to ours we decide to chose only among those parameters that were interpretable in a clear way, so that we were then able to put reasonable support bounds and informative priors on them.

4.2 Results on the full datasets

In this section we include all the results obtained from the analysis of real data. Results are divided according to the priors used: in Subsection 4.2.1 we assumed the uninformative scenario, with uniform priors for all the parameters. Table 3 lists the priors and the values assumed known and fixed. In Subsection 4.2.2 we show the results of the analysis in the informative scenario, when we assumed we have uniform priors over all the parameters except for p_{ICU} and π as specified in the main text and reported in Table 4.

Unknown parameters definition	Parameter	Distribution
Susceptibility	π	$\sim Unif(0, 1)$
Initial infection rate	λ_0	$\sim Unif(0, 0.000241)$
Exponential growth rate	ψ	$\sim Unif(0, 0.39)$
Over-dispersion	η	$\sim Unif(1, 100)$
Probability of ICU admission given infection	p_{ICU}	$\sim Unif(0, 1)$
**Parameters assumed known**	**Parameter**	**Value**
Rate of becoming infectious	σ	1
Rate of recovery	γ	0.5797
Mean time from infection to ICU admission	$\mu_{ICU\|E}$	5.708
Variance of time from infection to ICU admission	$\sigma^2_{ICU\|E}$	18.24
Population of 2012/13	$N_{2012/13}$	53,679,750
Population of 2013/14	$N_{2013/14}$	54,091,200
Population of 2014/15	$N_{2014/15}$	54,551,450

Table 3: Flat prior distributions and fixed parameters.

4.2.1 Results with uniform priors

To obtain these results we ran 3 independent chains of 1.1 millions iterations of the Metropolis Hastings algorithm explained in Section 3.1. Of these, the first 100,000 iterations were used adaptively to tune the algorithm (i.e. to estimate the mixing

Parameter	Distribution
π	$\sim LogNorm(\log\mu = \log(0.401), \log\sigma = 0.2)$ [9]
p_{ICU}	$\sim LogNorm(\log\mu = \log(0.000239), \log\sigma = 1)$ [10]

Table 4: Informative prior distributions from previous findings.

parameter ν) and the following 200,000 were discarded as burn-in period. Moreover, we used a thinning factor of 100, saving only one iteration every 100.

Figure 7 displays the chains of the 6 parameters of the system. Despite all the parameters showing convergence, the chains of some of them (namely π and ψ) are moving over a uniform distribution and they are severely correlated. For these reason we choose to run the algorithm for many iteration and with a very high thinning factor.

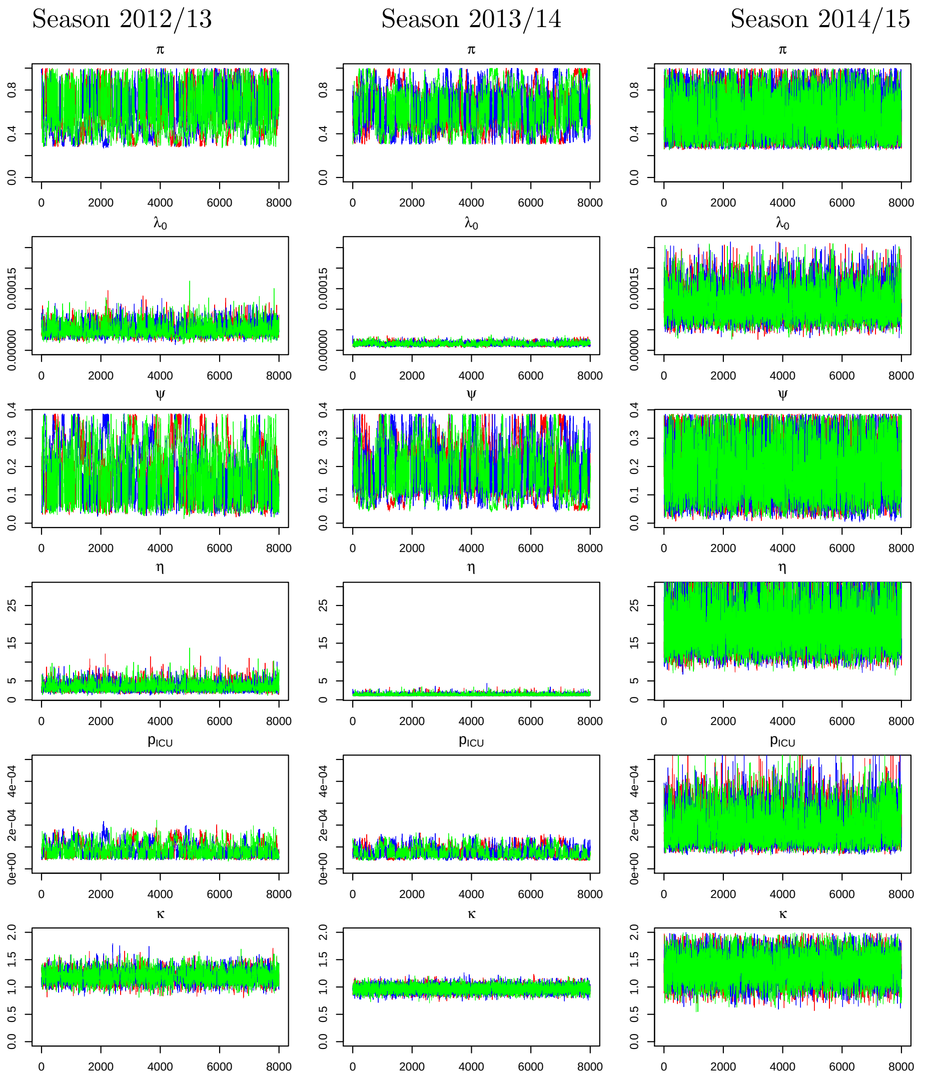

Figure 7: **MCMC of the parameters** The three independent chains are plotted here with three different colours (red, blue and green). The parameters are (in order, from top to bottom): the initial susceptibility π, the EGR ψ, the initial infection rate λ_0 the over-dispersion parameter η, the probability of ICU admission given infection p_{ICU} and the scaling parameter, κ. The results are derived from season 2012/13 (left column), season 2013/14 (centre) and season 2014/15 (right column).

The plots of the prior and the posterior distributions of all the parameters are reported in figure 8. Similarly, Figure 9 contains the plots of the prior and posterior

distribution of the other quantities of interest commented in the main text.

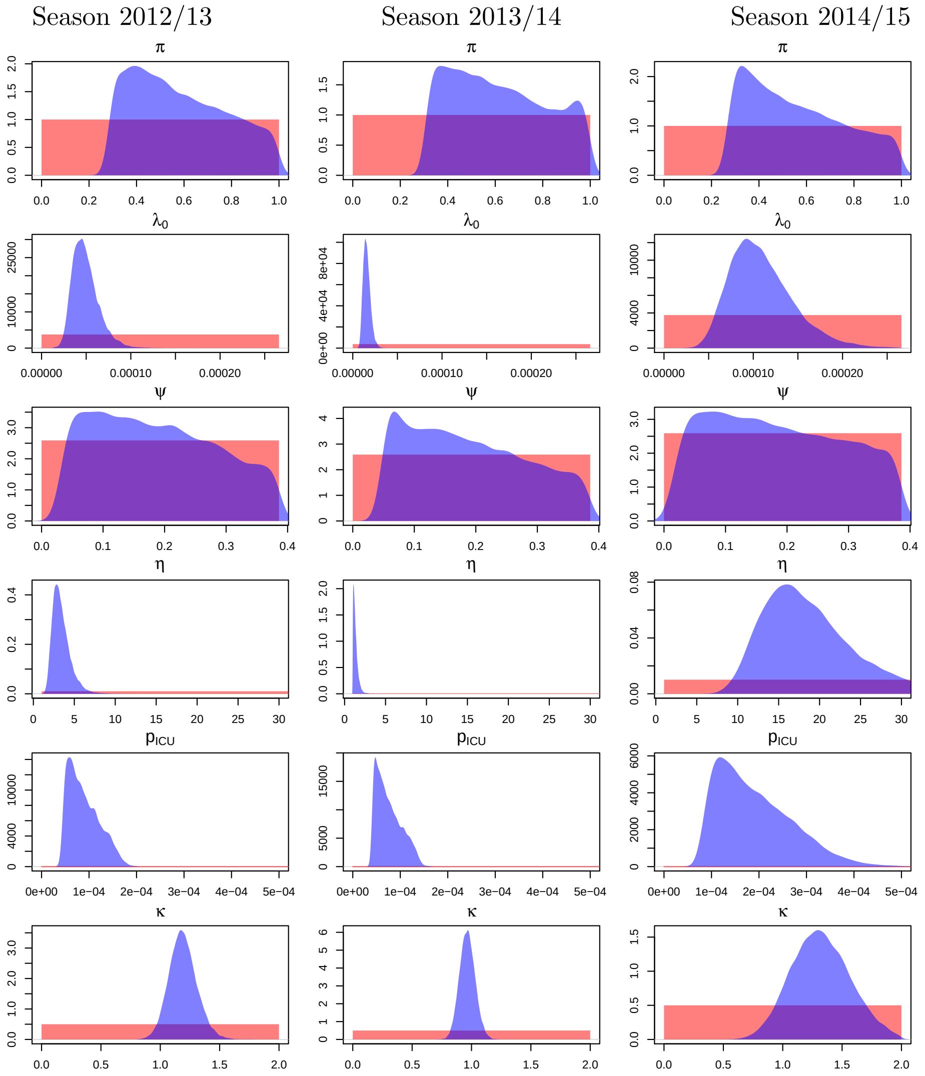

Figure 8: **Distributions of the parameters** Prior (red) and posterior (blue) distributions of the parameters. The parameters are (in order, from top to bottom): π, ψ, λ_0, η, p_{ICU} and κ. The results are derived from season 2012/13 (left column), season 2013/14 (centre) and season 2014/15 (right column).

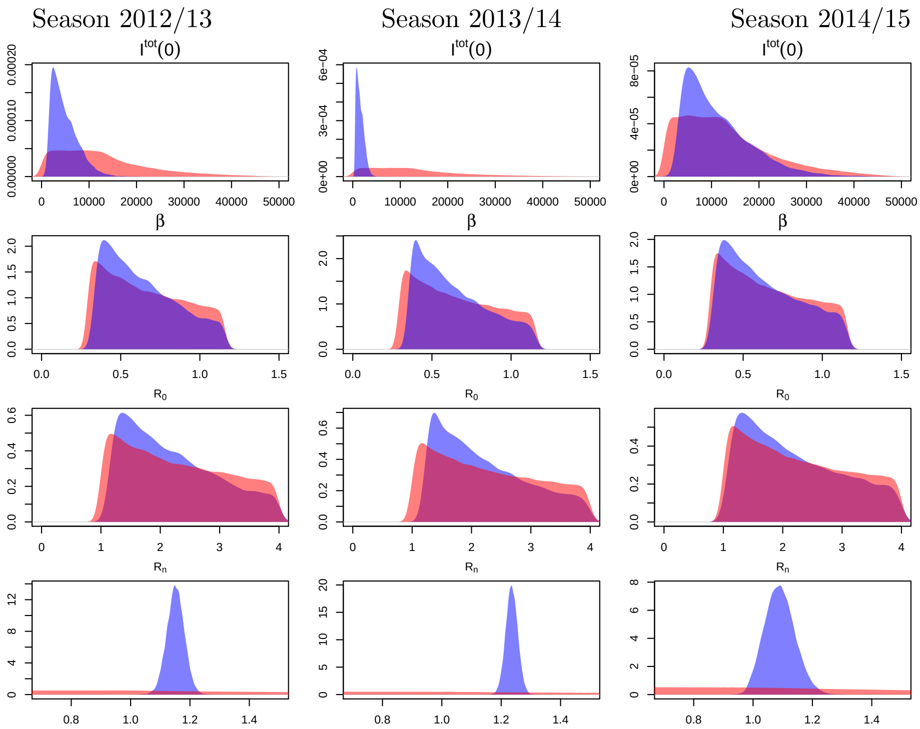

Figure 9: **Distributions of the epidemic metrics**. Prior (red) and posterior (blue) distributions of (from top to bottom): the total number of initial infectious I_0^{tot}, the basic transmission rate β, the basic reproduction number R_0 and the effective reproduction number R_n

Table 5 reports median and Credible Interval (CrI)s of all the parameters.

	2012/13	2013/14	2014/15
π	0.557 (0.298 - 0.969)	0.599 (0.323 - 0.978)	0.53 (0.28 - 0.968)
ψ	0.471 (0.271 - 0.842)	0.152 (0.091 - 0.248)	1.046 (0.549 - 1.921)
λ_0	0.18 (0.039 - 0.374)	0.179 (0.053 - 0.371)	0.178 (0.024 - 0.374)
η	3.204 (1.886 - 6.129)	1.251 (1.011 - 2.088)	17.947 (10.429 - 35.711)
p_{ICU}	0.083 (0.046 - 0.16)	0.07 (0.042 - 0.133)	0.175 (0.085 - 0.375)
κ	1.186 (0.974 - 1.437)	0.964 (0.84 - 1.099)	1.312 (0.864 - 1.82)
I_0^{tot}	4219.9 (1482.5 - 11510.8)	1386.2 (493 - 3328.4)	9568.6 (3052.3 - 28529)
β	0.6 (0.345 - 1.121)	0.598 (0.366 - 1.111)	0.597 (0.324 - 1.119)
R_0	2.071 (1.189 - 3.868)	2.063 (1.263 - 3.834)	2.058 (1.116 - 3.859)
R_n	1.152 (1.093 - 1.209)	1.235 (1.196 - 1.275)	1.089 (0.997 - 1.195)

Table 5: Posterior medians and 95% CrIs form the retrospective analysis of the ICU admissions over the three years considered.

4.2.2 Results with informative priors

We performed the same analysis assuming we had prior information on some of the parameters as reported in Table 4.

To obtain these results we ran 3 independent chains of 1.1 millions iterations of the Metropolis Hastings algorithm explained in Section 3.1. Of these, the first 100,000 iterations were used adaptively to tune the algorithm (i.e. to estimate the mixing parameter ν) and the following 200,000 were discarded as burn-in period. Moreover, we used a thinning factor of 100, saving only one iteration every 100.

Figure 10 displays the chains of the 6 parameters of the system. All the chains have now converged to a non-flat distribution.

The plots of the prior and the posterior distributions of all the parameters are reported in figure 11. Similarly, Figure 9 contains the plots of the prior and posterior distribution of the other quantities of interest.

The lack of information has been compensated by the introduction of prior of information. Due to the very high correlation that is affecting the system, by putting a prior on π and p_{ICU} we affect also other parameters, such as ψ, β and R_0. However, the posterior distributions of these parameters are driven by the prior distributions alone. As a signal of this problem the posterior distributions looks almost identical across the different seasons (i.e. with different data).

Table 6 reports median and CrIs of all the parameters.

	2012/13	2013/14	2014/15
π	0.4 (0.3 - 0.581)	0.403 (0.316 - 0.58)	0.406 (0.292 - 0.588)
λ_0	0.405 (0.242 - 0.681)	0.128 (0.082 - 0.201)	0.916 (0.508 - 1.601)
ψ	0.278 (0.167 - 0.373)	0.297 (0.188 - 0.379)	0.256 (0.147 - 0.364)
η	3.188 (1.889 - 6.039)	1.248 (1.012 - 2.113)	17.503 (10.323 - 33.765)
p_{ICU}	0.114 (0.077 - 0.162)	0.104 (0.071 - 0.137)	0.22 (0.138 - 0.358)
κ	1.184 (0.97 - 1.433)	0.966 (0.842 - 1.097)	1.318 (0.883 - 1.825)
I_0^{tot}	2603.2 (1318.1 - 5533.9)	787 (431 - 1558.3)	6408.2 (2928.1 - 14205.3)
β	0.835 (0.572 - 1.117)	0.888 (0.617 - 1.135)	0.778 (0.532 - 1.089)
R_0	2.882 (1.974 - 3.853)	3.065 (2.129 - 3.916)	2.684 (1.834 - 3.757)
R_n	1.153 (1.093 - 1.213)	1.235 (1.196 - 1.276)	1.089 (0.997 - 1.194)

Table 6: Posterior medians and 95% CrIs form the retrospective analysis of the ICU admissions over the three years considered.

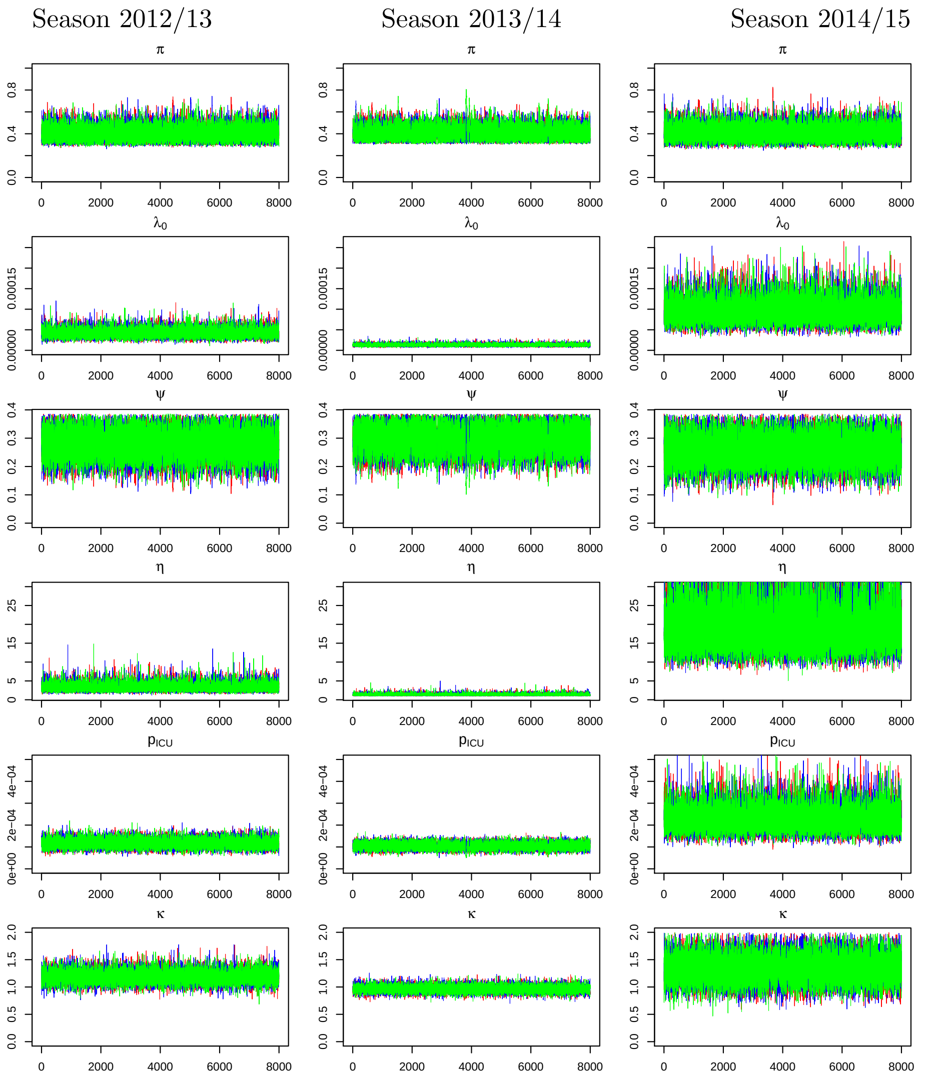

Figure 10: **MCMC of the parameters** The three independent chains are plotted here with three different colours (red, blue and green). The parameters are (in order, from top to bottom): π, ψ, λ_0, η, p_{ICU} and the scaling parameter, κ. The results are derived from season 2012/13 (left column), season 2013/14 (centre) and season 2014/15 (right column).

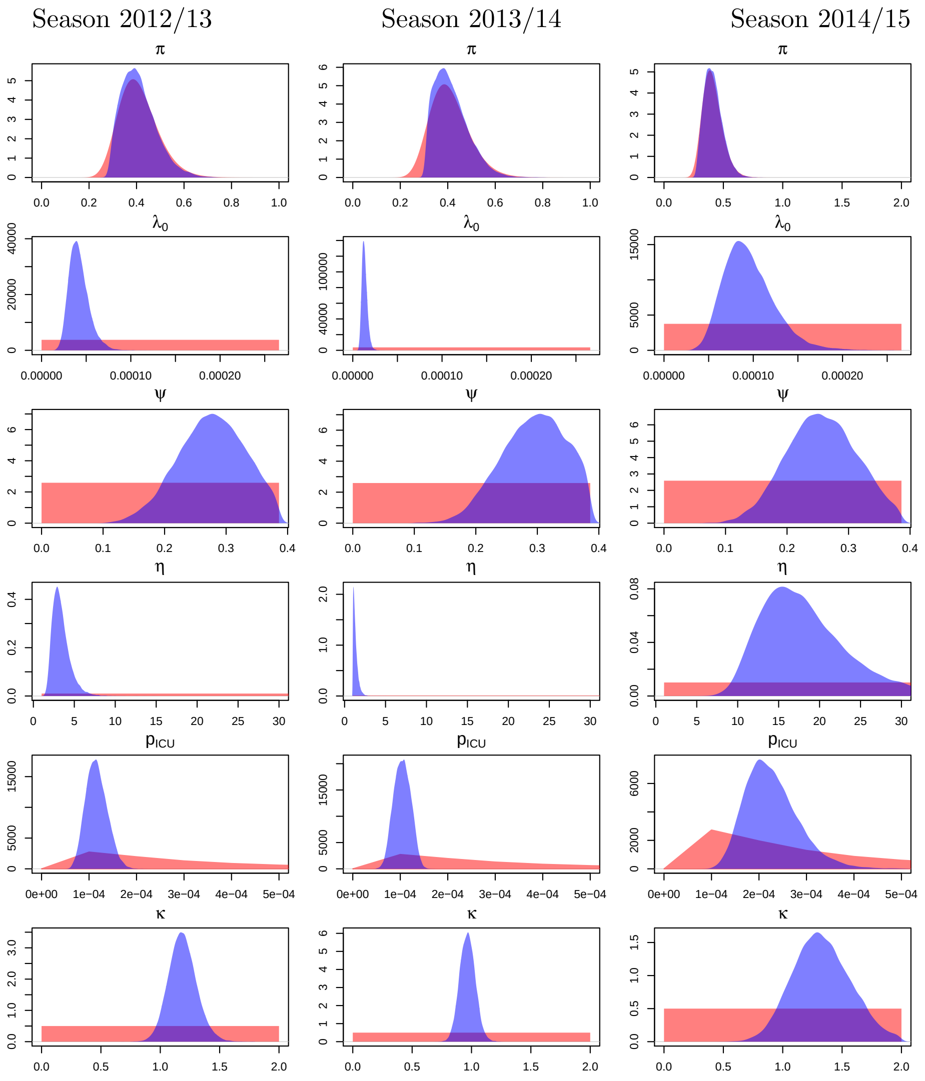

Figure 11: **Distributions of the parameters** Prior (red) and posterior (blue) distributions of the parameters. The parameters are (in order, from top to bottom): π, ψ, λ_0, η, p_{ICU} and κ. The results are derived from season 2012/13 (left column), season 2013/14 (centre) and season 2014/15 (right column).

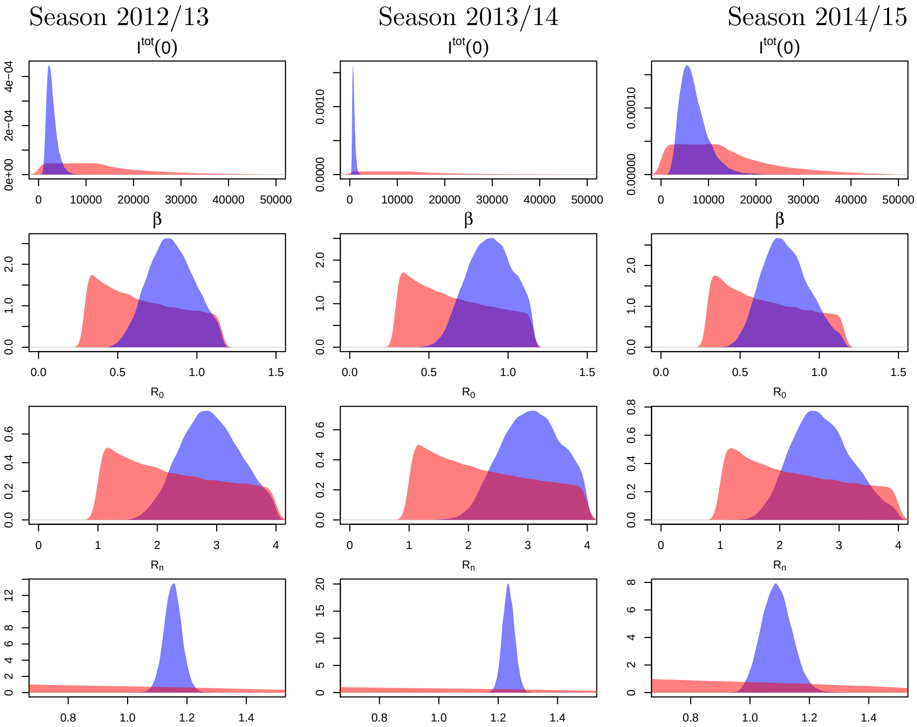

Figure 12: **Distributions of the epidemic metrics**. Prior (red) and posterior (blue) distributions of (from top to bottom): the total number of initial infectious I_0^{tot}, the basic transmission rate β, the basic reproduction number R_0 and the effective reproduction number R_n

Lastly, Figure 13, reports the posterior predictive distribution of the number of ICU admissions. There is no significant improvement compared to the same result within the uninformative scenario (Figure 5 of the main text).

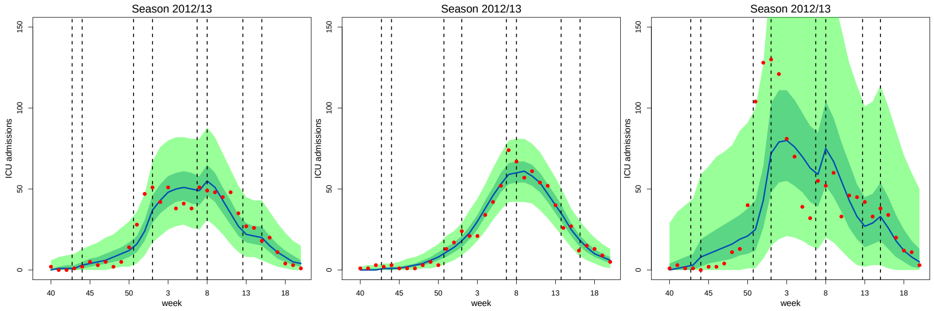

Figure 13: **Retrospective analysis.** Median (blue), 95 % CrI (light green) and quartile (dark green) of the posterior predictive distributions and observed values (red) of the weekly ICU/HDU admissions across seasons. The vertical dashed lines represent the breakpoints for the piecewise tranmsissibility β^*

4.3 Results on the datasets updated every five weeks

The sequential learning of the parameter via inclusion of more and more data has already been discussed in Subsection 3.2. For this reason here we report only the performance of the model at predicting data different times in the uninformative scenario for the sake of comparison with the result presented in figure 6 of the main text. This is reported in Figure 14 and it highlights the extreme need of informative prior information in order to obtain useful prediction.

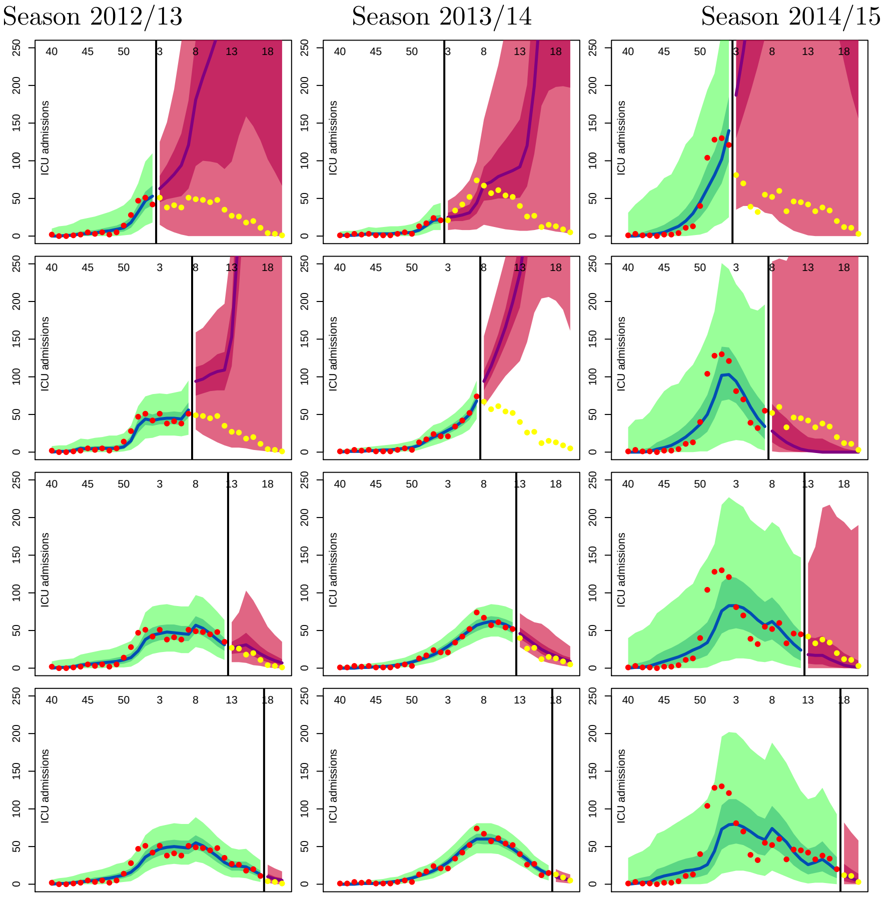

Figure 14: **Prospective analysis.** The black line displays the analysis time; the blue line and green shaded area represent median, quartile (dark green) and 95% CrIs (light green) of the posterior predictive distribution for the training dataset weeks. The pink area displays posterior quartiles (deep pink) and 95% CrIs (light pink) for the predicted future observations, and the purple line displays the median; the red dots are the training data and the yellow dots are the observations we have predicted.

5 Simulation of a pandemic

We simulated data on the number of hospital admissions during an epidemic. In this case, more data are collected: all the hospitals have to report the weekly number of admissions at all levels of care. We assumed the values of the parameters were fixed

to:

$$\begin{aligned} \pi &= 0.5 \\ \lambda_0 &= 0.0001 \\ \psi &= 0.2 \\ \eta &= 20 \\ p_H &= 0.002 \\ \kappa &= 1.5 \end{aligned} \tag{17}$$

where p_H is the probability of hospitalization given infection. We simulated the weekly hospital admissions over a season and this resulted in the counts plotted in Figure 15.

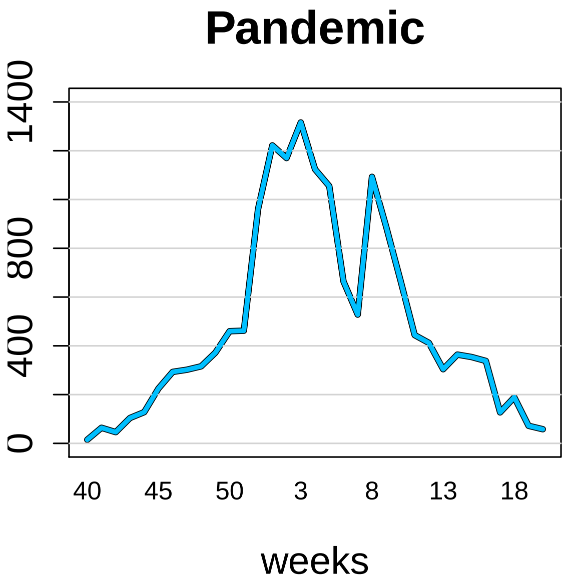

Figure 15: Simulated data in the case of a pandemic

5.1 Results on the full datasets

5.1.1 Results with uniform priors

To obtain these results we ran 3 independent chains of 1.1 millions iterations of the Metropolis Hastings algorithm explained in Section 3.1. Of these, the first 100,000 iterations were used adaptively to tune the algorithm (i.e. to estimate the mixing parameter ν) and the following 200,000 were discarded as burn-in period. Moreover, we used a thinning factor of 100, saving only one iteration every 100. The algorithm is run for less iteration then in the analysis of ICU data, because the computation time for each iteration increased due to the the higher values of the data.

Figure 16 displays the chains of the 6 parameters of the system. Despite all the parameters showing convergence, the chains of some of them (namely π and ψ) are moving over a uniform distribution and the chains are particularly constraint. For these reason we choose to run the algorithm for many iteration and with a very high thinning factor. This shows that, despite the number being higher in the case of a pandemic, the identifiability issue is not solved

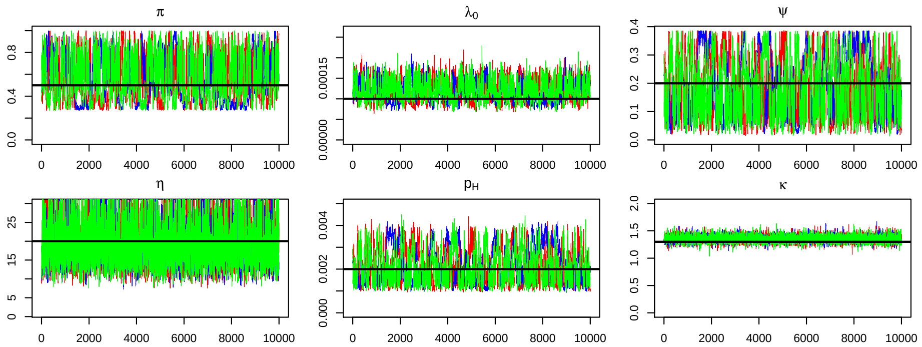

Figure 16: **MCMC of the parameters** The three independent chains are plotted here with three different colours (red, blue and green). The parameters are (in order, from top to bottom): the initial susceptibility π, the EGR ψ, the initial infection rate λ_0 the over-dispersion parameter η, the probability of hospital admission given infection p_H and the scaling parameter, κ. The block horizontal line denotes the values used to simulate the dataset.

The plots of the prior and the posterior distributions of all the parameters are reported in figure 17. Similarly, Figure 18 contains the plots of the prior and posterior distribution of the other quantities of interest.

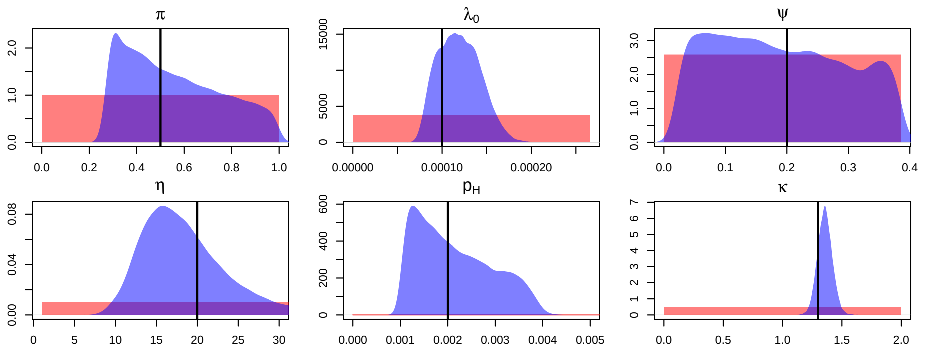

Figure 17: **Distributions of the parameters** Prior (red) and posterior (blue) distributions of the parameters. The parameters are (in order, from top to bottom): π, ψ, λ_0, η, p_H and κ. The block vertical line denotes the values used to simulate the dataset.

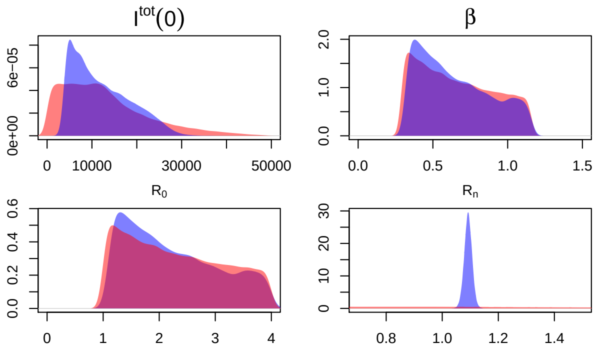

Figure 18: **Distributions of the epidemic metrics**. Prior (red) and posterior (blue) distributions of (from top to bottom): the total number of initial infectious I_0^{tot}, the transmission rate β, the basic reproduction number R_0 and the effective reproduction number R_n

Table 7 reports median and CrIs of all the parameters.

	Median (CrI)
π	0.523 (0.282 - 0.961)
λ_0	1.183 (0.804 - 1.662)
ψ	0.182 (0.028 - 0.375)
η	17.665 (10.677 - 32.4)
p_H	1.996 (1.072 - 3.747)
κ	1.356 (1.238 - 1.481)
I_0^{tot}	10721 (4104 - 25973.6)
β	0.605 (0.329 - 1.123)
R_0	2.087 (1.136 - 3.873)
R_n	1.092 (1.064 - 1.12)

Table 7: Posterior medians and 95% CrIs form the retrospective analysis of the Hospital admissions.

5.1.2 Results with informative priors

Figure 19 displays the chains of the 6 parameters of the system. Despite all the parameters showing convergence, the chains of some of them (namely π and ψ) are moving over a uniform distribution and the chains are particularly constraint.

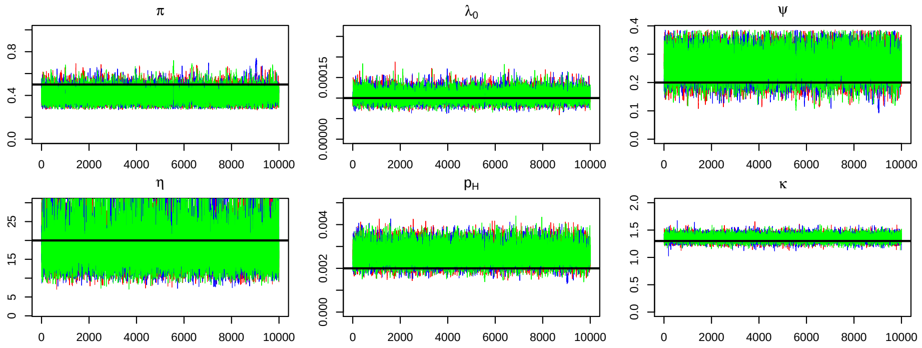

Figure 19: **MCMC of the parameters** The three independent chains are plotted here with three different colours (red, blue and green). The parameters are (in order, from top to bottom): π, ψ, λ_0, η, p_H and κ.

The plots of the prior and the posterior distributions of all the parameters are reported in figure 20. Similarly, Figure 21 contains the plots of the prior and posterior distribution of the other quantities of interest.

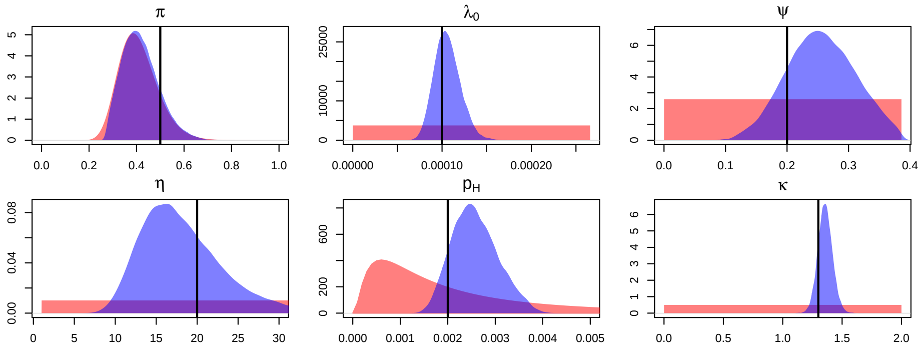

Figure 20: **Distributions of the parameters** Prior (red) and posterior (blue) distributions of the parameters. The parameters are (in order, from top to bottom): π, ψ, λ_0, η, p_H and κ. The block vertical line denotes the values used to simulate the dataset.

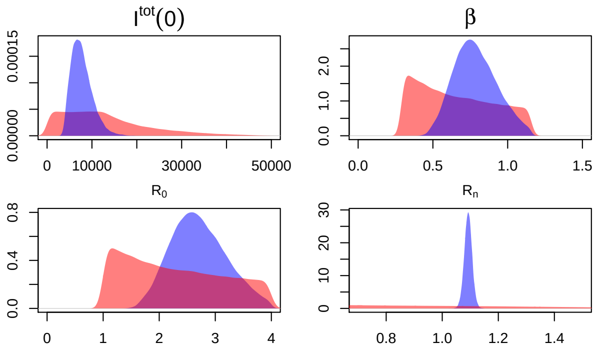

Figure 21: **Distributions of the epidemic metrics**. Prior (red) and posterior (blue) distributions of (from top to bottom): the total number of initial infectious I_0^{tot}, the basic transmission rate β, the basic reproduction number R_0 and the effective reproduction number R_n

Table 8 reports median and CrIs of all the parameters.

	Median (CrI)
π	0.409 (0.293 - 0.591)
λ_0	1.05 (0.802 - 1.37)
ψ	0.254 (0.148 - 0.362)
η	17.462 (10.574 - 31.958)
p_H	2.539 (1.744 - 3.596)
κ	1.357 (1.24 - 1.482)
I_0^{tot}	7412.1 (4260.8 - 13170)
β	0.774 (0.535 - 1.082)
R_0	2.671 (1.845 - 3.732)
R_n	1.092 (1.065 - 1.12)

Table 8: Posterior medians and 95% CrIs form the retrospective analysis of the Hospital admissions.

5.2 Results on the datasets updated every five weeks

We report here the predictive performance of the model when data arrive in "real time". Informative prior distributions on the parameters π and p_H.
Posterior predictive distributions are reported in Figure 22. The precision of the predictions have increased a lot, making the results more useful. On the other hand, predictions could be precise and wrong, as shown in the second panel, where we underestimate the future number of hospital admissions.

References

[1] Tom BDM, van Hoek AJ, Pebody R, McMenamin J, Robertson C, Catchpole M, De Angelis D. Estimating time to onset of swine influenza symptoms after initial novel A(H1N1v) viral infection. Epidemiol Infect. 2011;139(9):1418–1424. Available from: http://www.ncbi.nlm.nih.gov/pubmed/21087539. doi:10.1017/S0950268810002566.

[2] Akaike H. Akaikes information criterion. In: International Encyclopedia of Statistical Science. Springer; 2011. p. 25–25.

[3] Vynnycky E, White R. An introduction to infectious disease modelling. Oxford University Press; 2010.

[4] Wearing HJ, Rohani P, Keeling MJ. Appropriate models for the management of infectious diseases. PLoS Med. 2005;2(7):0621–0627. doi:10.1371/journal.pmed.0020174.

[5] Birrell PJ, Ketsetzis G, Gay NJ, Cooper BS, Presanis AM, Harris RJ, Charlett A, Zhang XS, White PJ, Pebody RG, et al. Bayesian modeling to unmask and predict influenza A/H1N1pdm dynamics in London. Proc Natl Acad Sci. 2011;108(45):18238–18243. doi:10.1073/pnas.1103002108.

[6] Robert C, Casella G. Introducing Monte Carlo Methods with R. Springer Science & Business Media; 2009.

[7] Sherlock C, Fearnhead P, Roberts GO. The Random Walk Metropolis: Linking Theory and Practice Through a Case Study. Stat Sci. 2010;25(2):172–190. Available from: http://projecteuclid.org/euclid.ss/1290175840. doi:10.1214/10-STS327.

[8] Gustafson P. Bayesian inference for partially identified models. Int J Biostat. 2010;6(2):17. Available from: http://www.scopus.com/inward/record.url?eid=2-s2.0-77950543899{&}partnerID=40{&}md5=3560eb2e5bac753d7ab038bab4699e9d. doi:17̊10.2202/1557-4679.1206.

[9] Hoschler K, Thompson C, Andrews N, Galiano M, Pebody R, Ellis J, Stanford E, Baguelin M, Miller E, Zambon M. Seroprevalence of influenza A(H1N1) pdm09 virus antibody, England, 2010 and 2011. Emerg Infect Dis. 2012;18(11):1894–1897. doi:10.3201/eid1811.120720.

[10] Presanis AM, Pebody RG, Birrell PJ, Tom BDM, Green RK, Durnall H, Fleming D, De Angelis D. Synthesising evidence to estimate pandemic (2009) A/H1N1 influenza severity in 2009-2011. Ann Appl Stat. 2014;8(4):2378–2403. doi:10.1214/14-AOAS775.

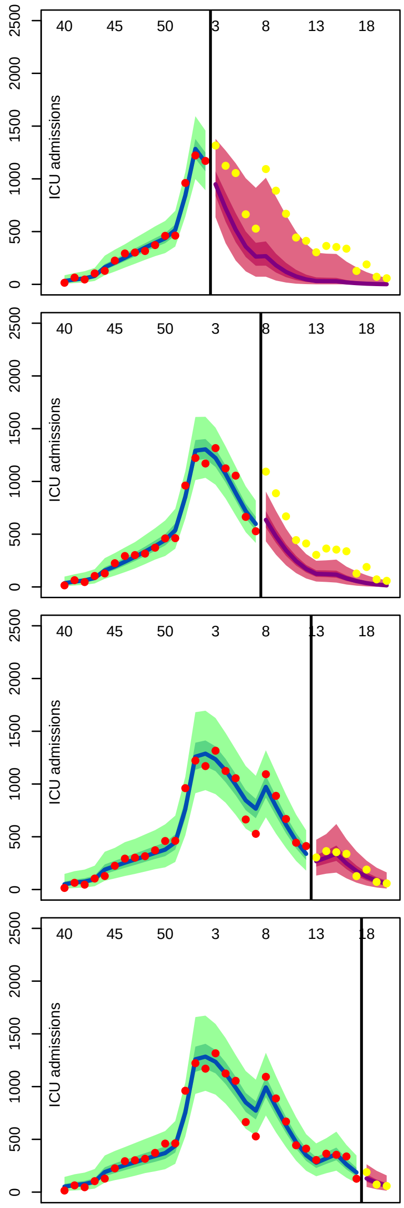

Figure 22: **Prospective analysis.** The black line displays the analysis time; the blue line and green shaded area represent median, quartile (dark green) and 95% CrIs (light green) of the posterior predictive distribution for the training dataset weeks. The pink area displays posterior quartiles (deep pink) and 95% CrIs (light pink) for the predicted future observations, and the purple line displays the median; the red dots are the training data and the yellow dots are the observations we have predicted.
